# Efficacy of bepotastine compared with hydroxyzine in preventing rituximab-induced infusion-related reactions in non-hodgkin lymphoma patients: a phase II, double-blind, multicenter, and randomized trial

**DOI:** 10.1007/s10147-026-03012-3

**Published:** 2026-04-16

**Authors:** Yumi Kitahiro, Hironobu Minami, Kazuhiro Yamamoto, Kimikazu Yakushijin, Keiji Kurata, Rina Sakai, Miki Saeki, Yuri Okazoe-Hirakawa, Kotaro Iida, Natsuko Murase, Isamu Harima, Naoko Kitamura, Kotaro Itohara, Tomohiro Omura, Takeshi Sugimoto, Hidetomo Takakura, Akihito Kitao, Masako Takahashi, Manabu Shimoyama, Mamoru Okuno, Ikuko Yano

**Affiliations:** 1 Department of Pharmacy, Kobe University Hospital, 7-5-2 Kusunoki-cho, Chuo-ku, Kobe, 650-0017 Japan; 2 Division of Medical Oncology/Hematology, Department of Medicine, Kobe University Hospital and Graduate School of Medicine, Kobe, Japan; 3 Department of Integrated Clinical and Basic Pharmaceutical Sciences, Faculty of Medicine, Dentistry and Pharmaceutical Sciences, Okayama University, Okayama, Japan; 4 Department of Hematology and Oncology, Kita-Harima Medical Center, Ono, Japan; 5 Department of Pharmacy, Kita-Harima Medical Center, Ono, Japan; 6 Department of Hematology and Oncology, Kohnan Medical Center, Kobe, Japan; 7 Department of Pharmacy, Kohnan Medical Center, Kobe, Japan

**Keywords:** Rituximab, Infusion-related reactions, Non-Hodgkin lymphoma, Bepotastine, Hydroxyzine, Drowsiness

## Abstract

**Background:**

This study evaluated the efficacy of hydroxyzine and bepotastine, first- and second-generation H1 receptor antagonists (H1RA), as pretreatments to prevent infusion-related reactions (IRRs) during the initial rituximab infusion in patients with non-Hodgkin lymphoma.

**Methods:**

In this double-blind, multicenter, randomized phase II study, 40 patients received hydroxyzine or bepotastine with acetaminophen 30 min before rituximab infusion. Primary endpoint was incidence of ≥ grade 2 IRRs based on the National Cancer Institute Common Terminology Criteria for Adverse Events. Secondary endpoints included IRRs severity, time to IRR onset, and H1RA-induced drowsiness.

**Results:**

Incidence of ≥ grade 2 IRRs was 52.4% and 31.6% for the hydroxyzine (n = 21) and bepotastine (n = 19) groups, respectively (*P* = 0.184). Distribution of initial and maximum IRR grades in the two groups was not statistically significant (*P* = 0.846 and 0.555). Median time (range) to IRR onset in the two groups was 67 (12–112) and 62 (10–119) min, respectively (*P* = 0.981). Median visual analog scale score (range and 75th percentile) for drowsiness was 37 (0–100, 46) and 12 (0–100, 29) mm in the two groups, respectively (*P* = 0.138). Incidence of ≥ grade 2 IRRs in the absence of bone marrow infiltration was 43.8% and 14.3% in the two groups, respectively (*P* = 0.118), and no group differences were observed with bone marrow infiltration.

**Conclusions:**

Bepotastine did not show significant superiority over hydroxyzine in preventing rituximab-induced IRRs due to the small sample size. Nevertheless, this exploratory study provides insight for further confirmatory studies.

**Trial registration number and date of registration:**

jRCTs051220169; February 14, 2023.

## Introduction

Rituximab is an anti-CD20 monoclonal antibody that is an essential component of most chemotherapeutic regimens for B-cell malignancies. It is often combined with cyclophosphamide, doxorubicin, vincristine, and prednisone (R-CHOP) [1]; however, infusion-related reactions (IRRs), such as fever, pruritus, rash, and anaphylactoid symptoms, frequently occur within 24 h of the initial rituximab infusion [2, 3]. These side effects are reported to occur in approximately 80% of patients receiving rituximab for the first time [4]. Although the precise pathogenesis of IRRs is unclear, it is usually affected by the rate of infusion, suggesting the possibility of a non-allergic mechanism and a role for inflammatory cytokines, such as interleukin (IL)-6 and tumor necrosis factor (TNF)-α [5]. Therefore, it is recommended that patients take an antipyretic analgesic and an antihistamine H1 receptor antagonist (H1RA) before rituximab infusion to ameliorate or prevent IRRs [6]. In clinical settings, first-generation H1RAs, which have been confirmed the efficacy in phase I and II clinical trials, are frequently administered [7, 8]; however, IRRs are still common despite the use of these premedications [2]. In addition, first-generation H1RAs have potent central nervous system effects, causing drowsiness during treatment. Moreover, the typical first-generation H1RAs, such as diphenhydramine and d-chlorpheniramine, exert an anticholinergic effect and are contraindicated for patients with occlusive glaucoma or benign prostatic hyperplasia [9].

Recently, a retrospective case-control study suggested the superiority of the second-generation H1RA bepotastine over diphenhydramine. The incidence of IRRs was 9.8% in the bepotastine group (n = 92), which was significantly lower compared with 30.2% in the diphenhydramine group (n = 96; *P* < 0.001) [10]. In addition, the second-generation H1RA may be superior to first-generation H1RAs in terms of milder drowsiness [11]; however, these observations were made in retrospective studies, and IRRs were evaluated based on physician’s subjective assessments [10]. These limitations may have introduced bias; thus, second-generation H1RAs should be compared with first-generation H1RAs in a prospective randomized study.

In this study, we conducted a prospective, double-blind, randomized study to evaluate the efficacy and safety of the second-generation H1RA bepotastine compared with those of the first-generation H1RA hydroxyzine during initial rituximab infusion in patients with non-Hodgkin lymphoma (NHL). Of the first-generation H1RAs, hydroxyzine was selected as a control because it can be used in patients with angle-closure glaucoma or prostatic hyperplasia. The primary objective of this study was to determine the incidence of grade 2 or higher IRRs in each of the hydroxyzine and bepotastine groups based on the National Cancer Institute Common Terminology Criteria for Adverse Events (CTCAE) version 5.0 [12] within 4 h following the initial infusion of rituximab. The secondary objectives were to evaluate the severity of the initial and maximum IRRs, time to initial IRR onset, and the incidence of adverse events, including drowsiness attributed to H1RA, based on the value of the visual analog scale (VAS), within the same 4-h period post-rituximab infusion.

## Materials and methods

### Study design

This study was a double-blind, active-controlled, multicenter, randomized phase II study [13]. It was designed according to CONSORT statement guidelines [14].

### Inclusion and exclusion criteria

This study included individuals aged ≥ 18 years at the time of consent, diagnosed with NHL and who received their first rituximab infusion as a standalone agent on day 1 of each regimen: cyclophosphamide, doxorubicin, vincristine, and prednisolone (R-CHOP); polatuzumab vedotin, cyclophosphamide, doxorubicin, and prednisolone (Pola-R-CHP); or bendamustine (BR). Patients were excluded if they had received orally or intravenously administration of short half-life (T_1/2_) antipyretic analgesics (e.g., acetaminophen, ibuprofen, and celecoxib) and corticosteroids within two days, long T_1/2_ antipyretic analgesics (e.g., oxaprozin, piroxicam, and naproxen) within 10 days, and H1RAs within five days, as well as obinutuzumab before rituximab treatment. Other exclusion criteria included poor renal function as indicated by creatinine clearance (Ccr) < 50 mL/min, liver dysfunction as determined by a Child–Pugh score C, severe interstitial pneumonitis, porphyria, known or possible pregnancy, and known allergies to the drugs used in this study.

### Intervention

The drugs were prepared by the Department of Pharmacy, Kobe University Hospital. To maintain the blind nature of the study, a non-blind pharmacist prepared the study drugs by placing either a 25 mg hydroxyzine capsule or a 10 mg bepotastine tablet inside an opaque capsule (Size #2). Patients were randomly selected to receive either drug. Randomization was stratified by the presence or absence of bone marrow infiltration. Patients received a combination of the study drug and acetaminophen tablets (400 mg) 30 min before rituximab infusion, with an allowance window of up to 60 min. Rituximab was administered intravenously following pre-specified schedules at each institution. Typically, the infusion was initiated at a rate of 50 mg/h and escalated by 50 mg/h every 30 min, up to a maximum rate of 400 mg/h.

### Endpoints

The primary endpoint was grade 2 or higher IRRs within 4 h following the initiation of rituximab infusion. Secondary endpoints included: (1) the severity of the initial and maximum IRRs within 4 h after initiating rituximab infusion, (2) time to the initial IRR onset during the 4-h period, and (3) the incidence of adverse events, including drowsiness attributed to H1RA, using the value of visual analog scale (VAS). The infusion time of rituximab in each group was compared as an exploratory endpoint.

### Assessments

#### Evaluation of IRRs

The severity of the IRRs was evaluated using the grading system of the CTCAE (version 5.0), based on the category of “infusion-related reaction.” IRR was assessed by physicians registered as investigators of the study. Each investigator was an established oncologist, trained by the principal investigator on IRR grading prior to the study, and followed standardized instructions. In the event of an IRR, standard rescue care protocols specified by each institution were implemented.

#### Evaluation of adverse events, including drowsiness caused by H1RA

Patients reported the severity of drowsiness using a VAS ranging from 0 mm (awake) to 100 mm (asleep), which was assessed 90 ± 15 min after ingesting the study drug. Assessment time was based on the average time to reach maximum plasma concentration (T_max_) for hydroxyzine and bepotastine following oral administration (approximately 126 and 72 min, respectively) [15, 16]. Other adverse events related to the study drug were also assessed.

### Statistics

#### Sample size calculation

The study included 40 participants, with 20 each allocated to the hydroxyzine and bepotastine groups. The primary objective was to assess the incidence of ≥ grade 2 IRRs for the two groups according to the CTCAE (version 5.0) within 4 h after initiating rituximab infusion. In our retrospective exploratory study at Kobe University Hospital, the incidence of ≥ grade 2 IRRs was 8/30 (26.7%) in NHL patients who received hydroxyzine before their first rituximab infusion. In contrast, a previous report on a retrospective study observed an incidence of ≥ grade 2 IRRs of 3/33 (9.1%) in patients treated with bepotastine [17]. Therefore, we conservatively estimated the incidence of grade 2 or higher IRRs as 25% for hydroxyzine and 10% for bepotastine. This assumption implies a 15% difference in the incidence between the two groups. For 20 patients in each group, the calculated widths for the 90% and 95% confidence intervals (CIs) for the difference between the two groups were 38.8% (CI: − 4.4% to 34.4%) and 46.2% (CI: − 8.1% to 38.1%), respectively.

### Statistical methods

The difference in IRR incidence between the hydroxyzine and bepotastine groups for the full analysis set (FAS) and the per-protocol set (PPS) was determined by a Pearson’s chi-square test. Their respective 90% and 95% CIs were evaluated by Clopper–Pearson CIs. FAS consisted of patients who were randomized and who completed the 4-h evaluation. PPS consisted of patients who were treated and evaluated without major protocol violations. Differences in the severity of the initial and maximum IRRs between hydroxyzine and bepotastine were determined using Fisher’s exact test. The time to initial IRRs onset, total rituximab infusion, and the VAS value indicating drowsiness caused by H1RAs are presented as the median and range for each group and compared by the Mann–Whitney U test. The safety of the study drug was assessed in all patients who received the study drug (safety analysis set; SAS). The analyses were performed using R software (R Foundation for Statistical Computing, version 2.8.0).

## Results

### Patient characteristics

**Fig. 1:**
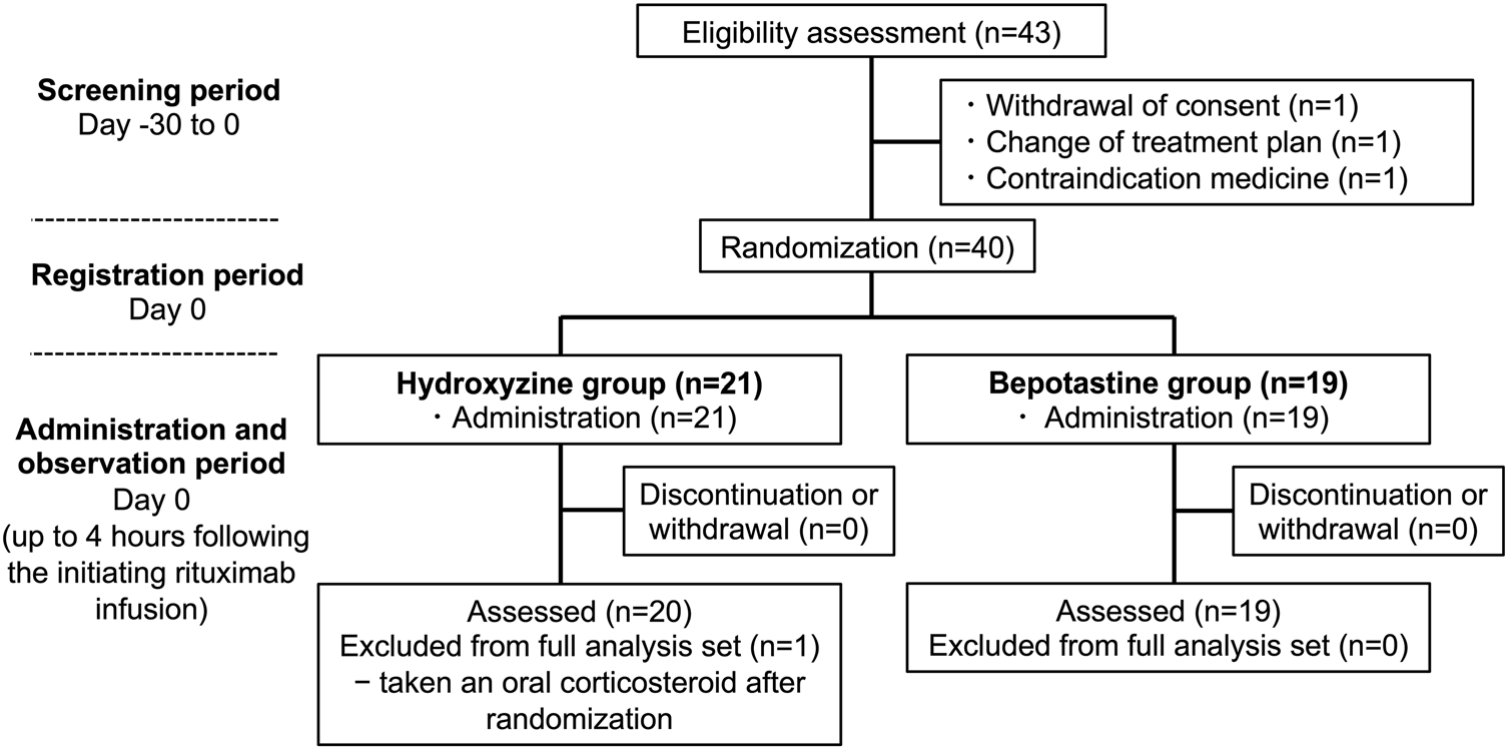
CONSORT flowchart showing the study design

Figure 1 shows the CONSORT flowchart of the study. A total of 43 patients signed an informed consent form between May 2, 2023 and December 26, 2024. After excluding three patients, 40 patients were enrolled and randomized to the hydroxyzine group (n = 21) or the bepotastine group (n = 19). All randomized patients were included in this study as FAS and SAS (n = 40). PPS included 39 patients, excluding one patient in the hydroxyzine group who took an oral corticosteroid after randomization. The patient characteristics are listed in Table 1. All randomized patients received rituximab at a dose of 375 mg/m^2^. None required modification or withholding of the planned escalation of the infusion rate.

**Table 1:** Patient characteristics

	Hydroxyzine (n = 21)	Bepotastine (n = 19)
Age, years^a^	66 (54–85)	69 (38–84)
Sex, Male, n (%)	15 (71.4)	10 (52.6)
BSA (m^2^)^a^	1.68 (1.33–2.19)	1.66 (1.41–1.81)
Child–Pugh classification, grade A/B, n (%)	20 (95.2)/1 (4.8)	17 (89.5)/2 (10.5)
ECOG PS score, n (%) 0 1 ≥ 2	6 (28.6) 14 (66.7) 1 (4.7)	5 (26.3) 11 (57.9) 3 (15.8)
B symptoms (+), n (%)	2 (9.5)	2 (10.5)
I II III IV	5 (23.8) 6 (28.6) 5 (23.8) 4 (19.0) 6 (28.6)	5 (26.3) 5 (26.3) 1 (5.3) 3 (15.8) 10 (52.6)
Bone marrow infiltration (+), n (%)	8 (38.1)	8 (42.1)
Ann Arbor Staging, n (%)		
LDH > 222 U/L, n (%)		
sIL-2 > 2,000 U/mL, n (%)	5 (23.8)	7 (36.8)
Non-Hodgkin lymphoma, n (%)		
Diffuse large B-cell lymphoma	15 (71.4)	12 (63.2)
Follicular lymphoma	4 (19.0)	4 (21.1)
Mantle cell lymphoma	1 (4.8)	1 (5.3)
Indolent lymphoma (subtype unknown)	1 (4.8)	1 (5.3)
Splenic B-cell marginal zone lymphoma	0	1 (5.3)
Regimen, n (%) R-CHOP Pola-R-CHP BR	11 (52.4) 5 (23.8) 5 (23.8) 21 (100)	11 (57.9) 1 (5.3) 7 (36.8) 19 (100)
Rituximab biosimilar, n (%)	628 (490–820)	607 (500–670)
Dosage of rituximab (mg)^a^		

^a^ Data are presented as the median (range)

*BR* prednisolone and rituximab plus bendamustine, *BSA* body surface area, *ECOG PS* performance status defined by The Eastern Cooperative Oncology Group, *R-CHOP* rituximab, cyclophosphamide, doxorubicin, vincristine, and prednisone, *Pola-R-CHP* polatuzumab vedotin combined with rituximab, cyclophosphamide, doxorubicin, and prednisone

### Primary analysis

**Fig. 2:**
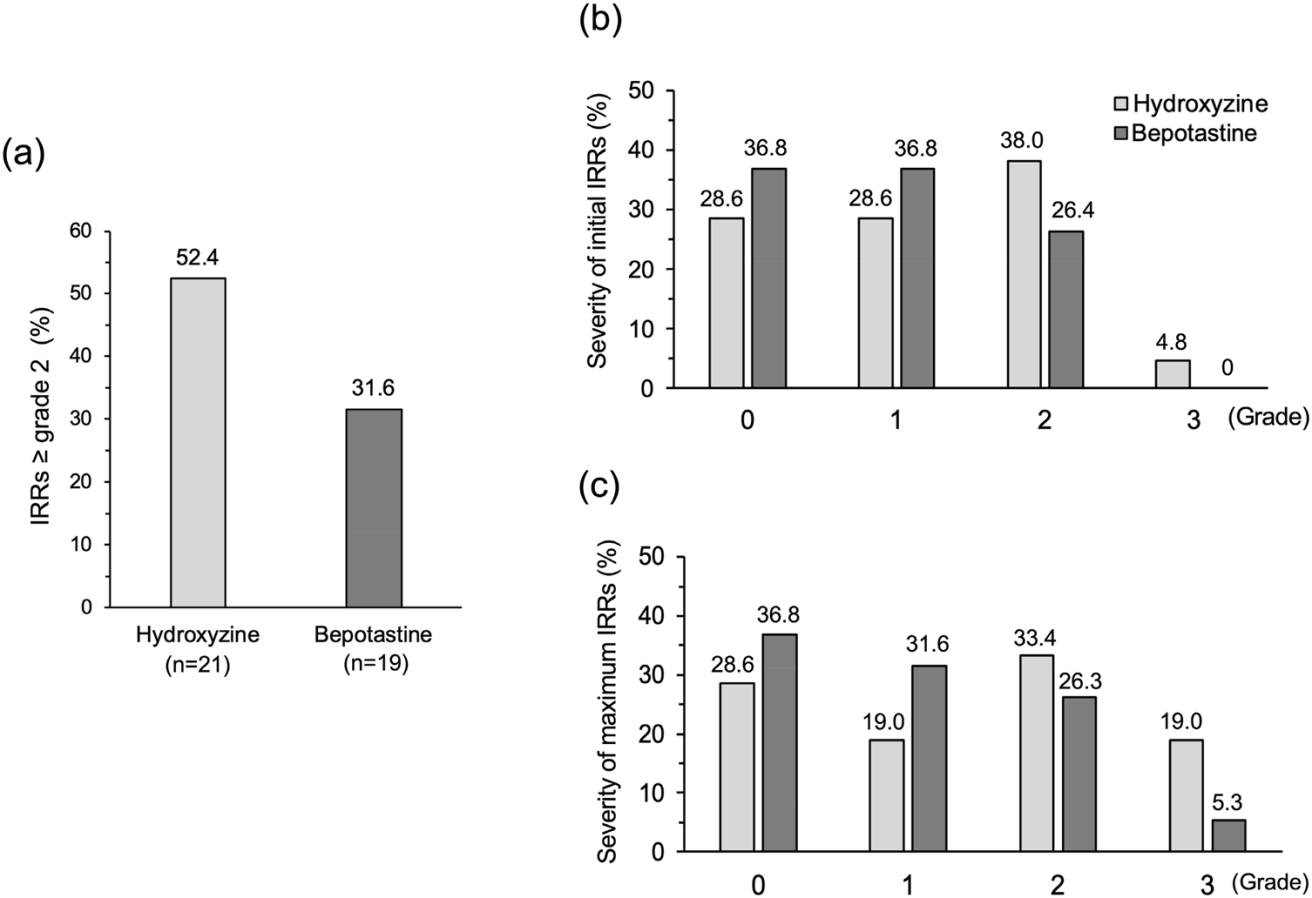
IRR occurrence within 4 h of rituximab infusion in the hydroxyzine and bepotastine groups based on FAS (**a)** Incidence of ≥ grade 2 IRRs to rituximab (**b)** Severity of initial IRRs (**c)** Severity of maximum IRRs. *IRRs* infusion-related reactions, *FAS* full analysis set

The primary endpoint results are shown in Fig. 2a. The total incidence of ≥ grade 2 IRRs in the hydroxyzine (n = 21) and bepotastine (n = 19) groups was 11 (52.4%) and 6 (31.6%), respectively, whereas the difference between the groups was 20.8% (*P* = 0.184; 90% and 95% CIs; − 4.3% to 45.9% [width 50.2%] and − 9.1% to 50.7% [width 59.8%], respectively). Because only one patient was excluded from the FAS, the result for PPS was nearly identical to that of FAS. The total incidence of ≥ grade 2 IRRs in the hydroxyzine (n = 20) and bepotastine (n = 19) groups was 11 (55.0%) and 6 (31.6%), respectively, whereas the difference between the two groups was 23.4% (*P* = 0.140; 90% and 95% CIs: − 1.9% to 48.8% [width 50.7%] and − 6.8% to 53.6% [width 60.4%], respectively).

### Secondary analyses

#### Severity of the initial IRRs within 4 h of rituximab infusion

The incidence for each grade of the initial IRRs in the FAS is shown in Fig. 2b; however, the difference between the two groups was not statistically significant (*P* = 0.846).

#### Severity of the maximum IRRs within 4 h of rituximab infusion

The incidence for each grade of the maximum severity in FAS was shown in Fig. 2c; however, there were no significant differences between the two groups (*P* = 0.555).

#### Time to initial IRRs onset within 4 h of rituximab infusion

**Fig. 3:**
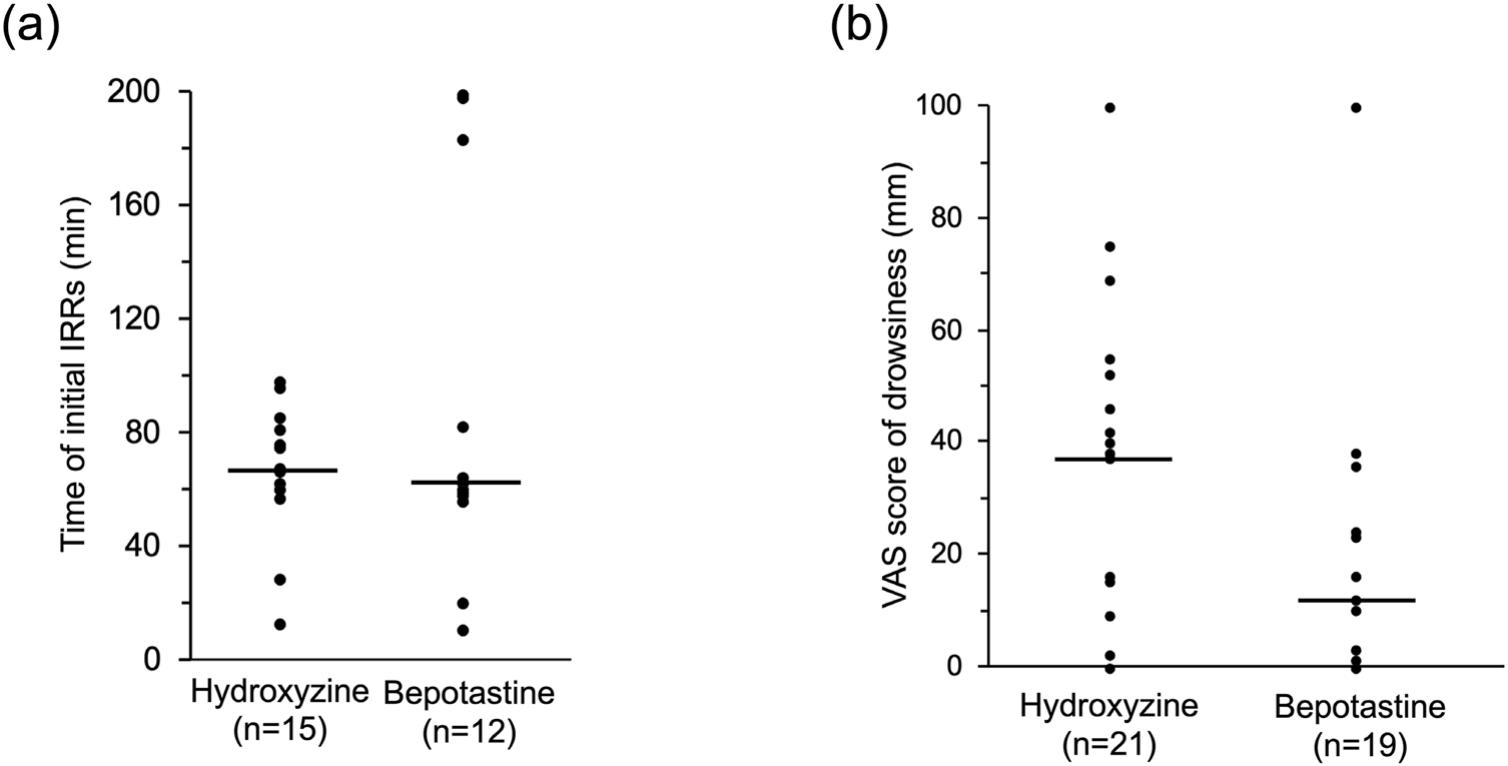
Onset time of the initial IRRs following rituximab infusion (**a**) and VAS score of H1RA-induced drowsiness (**b**). The bar shows the median value for each group. *IRRs* infusion-related reactions, *VAS* visual analog scale

The median time to initial IRR onset after the initiation of rituximab infusion (range) was 67 (12–112) min in the hydroxyzine group and 62 (10–119) min in the bepotastine group, whereas the difference between the groups was 5 min (*P* = 0.981) (Fig. 3a). The median of the total rituximab infusion time (range) was 215 (186–294) min in the bepotastine group, which was significantly shorter compared with 249 (192–348) min in the hydroxyzine group (*P* = 0.025). Other symptoms observed for the initial IRRs are listed in Table 2. Ten patients (47.6%) experienced any grade of chills in the hydroxyzine group, whereas eight patients (42.1%) experienced any grade of fever in the bepotastine group.

**Table 2:** Symptoms of infusion-related reactions

n (%)	Hydroxyzine (n = 21)		Bepotastine (n = 19)	
	All grade	≥ grade 2	All grade	≥ grade 2
Fever	4 (19.0)	3 (14.3)	8 (42.1)	3 (15.8)
Chills	10 (47.6)	7 (33.3)	4 (21.1)	3 (15.8)
Nausea/Vomiting	0	0	1 (5.3)	1 (5.3)
Sore throat	3 (14.3)	1 (4.8)	0	0
Pruritus	2 (9.5)	2 (9.5)	0	0
Rash	2 (9.5)	2 (9.5)	0	0
Rhinorrhea	1 (4.8)	1 (4.8)	2 (10.5)	2 (10.5)
Cough	0	0	1 (5.3)	1 (5.3)
Dyspnea	0	0	1 (5.3)	1 (5.3)
Hypoxia	1 (4.8)	1 (4.8)	0	0
Diaphoresis	1 (4.8)	1 (4.8)	0	0
Headache	0	0	1 (5.3)	1 (5.3)
Hypertension	0	0	1 (5.3)	1 (5.3)
Tremor	0	0	1 (5.3)	1 (5.3)
Pain	0	0	1 (5.3)	1 (5.3)
Hyperemia of eyes	1 (4.8)	1 (4.8)	0	0
Edema face	0	0	1 (5.3)	1 (5.3)

#### Evaluating drowsiness caused by H1RA

The median VAS scores (range and 75th percentile) for drowsiness by hydroxyzine and bepotastine were 37 (0–100, 46) mm and 12 (0–100, 29) mm, respectively. The difference between the two groups was 25 mm (*P* = 0.138) (Fig. 3b).

#### Evaluation of a stratification factor

**Fig. 4:**
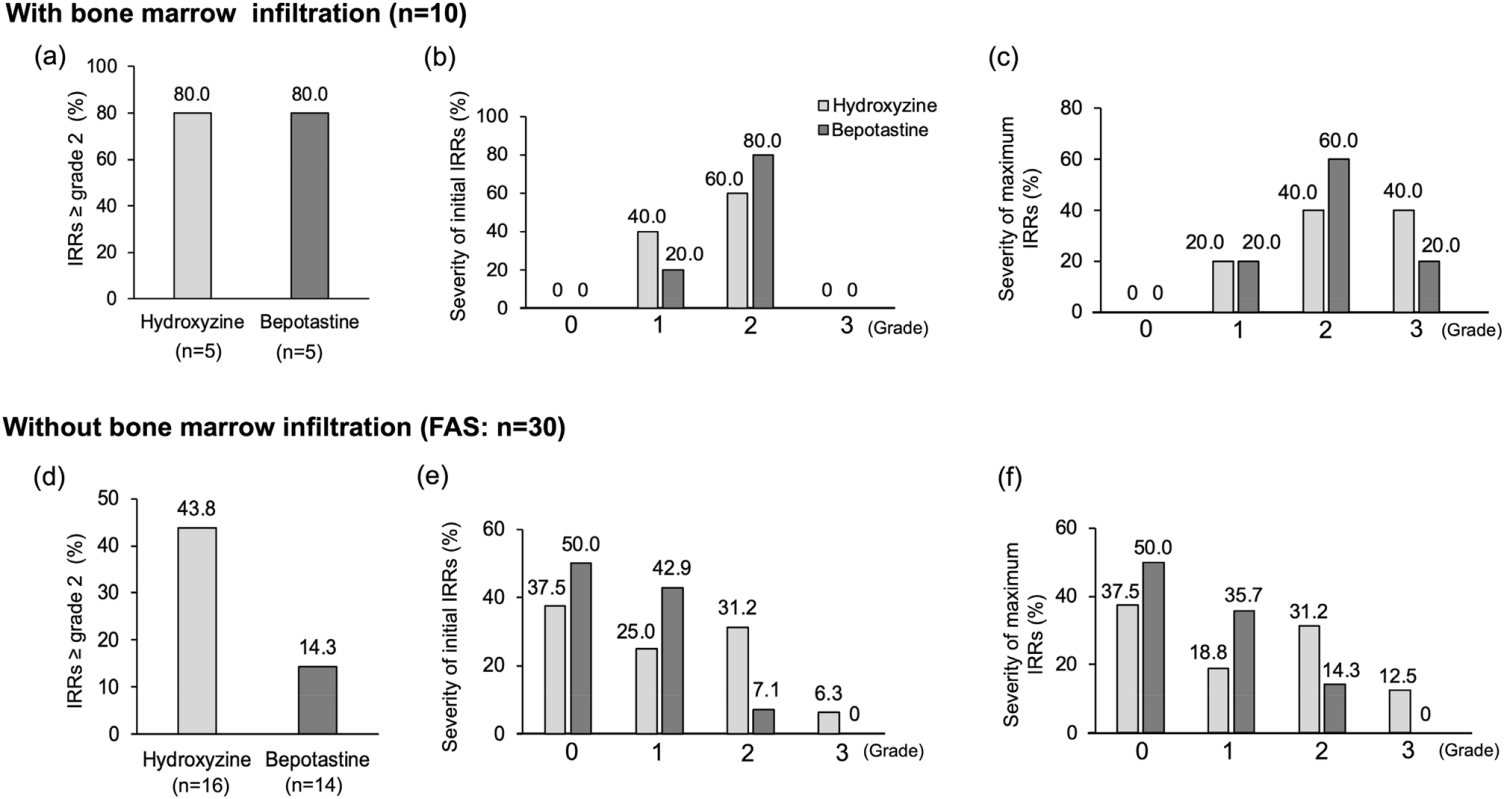
IRR occurrence within 4 h of rituximab infusion in the presence or absence of bone marrow infiltration (**a**) Incidence of ≥ grade 2 IRRs to rituximab with bone marrow infiltration (**b**) Severity of initial IRRs with bone marrow infiltration (**c**) Severity of maximum IRRs with bone marrow infiltration (**d**) Incidence of ≥ grade 2 IRRs associated with rituximab without bone marrow infiltration in FAS (**e**) Severity of initial IRRs without bone marrow infiltration in FAS (**f**) Severity of maximum IRRs without bone marrow infiltration in FAS. *IRRs* infusion-related reactions, *FA*S full analysis set

Infiltration of lymphoma cells into the bone marrow is a known risk factor for IRRs; therefore, randomization was stratified by bone marrow infiltration in the present study. We examined the potential association between IRR occurrence and bone marrow infiltration (Fig. 4). For patients with bone marrow infiltration (n = 10), the incidence of ≥ grade 2 IRRs was 4 out of 5 patients in both the hydroxyzine and bepotastine groups (Fig. 4a). In contrast, in the absence of bone marrow infiltration, the total incidence of ≥ grade 2 IRRs in the hydroxyzine group (7 out of 16 patients, 43.8%) was higher compared with that of the bepotastine group (2 of 14 patients, 14.3%), although the difference was not statistically significant (*P* = 0.118, Fig. 4d).

#### Safety assessment

No adverse reactions related to the study drugs were observed in either the hydroxyzine (n = 21) or bepotastine (n = 19) group during the study period.

## Discussion

To our knowledge, this is the first double-blind, randomized controlled trial comparing the effects of the first-generation H1RA hydroxyzine and the second-generation H1RA bepotastine for the prevention of IRRs induced by rituximab infusion. Our results indicated that the total incidence of ≥ grade 2 IRRs in the bepotastine group was 20.8% lower compared with that in the hydroxyzine group; however, the difference was not statistically significant because of the small sample size.

In clinical practice, first-generation H1RAs are widely used for preventing IRRs. Diphenhydramine and hydroxyzine are widely used as premedications for rituximab; however, no previous studies have compared the effects of two drugs. Due to the similar T_max_ values of hydroxyzine (2.1 ± 0.4 h) and diphenhydramine (2.3 ± 0.2 h), their prophylactic effects against IRRs are expected to be comparable; thus, hydroxyzine was selected as the first-generation H1RA for this study.

In a previous report, T_max_ of TNF-α and IL-6, which are putative mechanisms of IRRs, was 90 min following rituximab infusion [18], and severe IRRs were manifested 30 to 120 min after the initiation of rituximab infusion [6]. The onset of the pharmacological effects of H1RA may be determined by their T_max_ and T_1/2_ values, particularly T_max_ [19]. These characteristics may also influence their efficacy in suppressing IRRs. Considering that the mean T_max_ values for bepotastine and hydroxyzine are 1.2 and 2.1 h, respectively [15, 16], bepotastine and hydroxyzine likely reach their T_max_ at approximately 42 and 96 min, respectively, following the initiation of rituximab infusion in the present study, as the drugs were administered 30 min before rituximab. The median time to initial IRRs onset was 62 and 67 min for bepotastine and hydroxyzine, respectively. These results suggest that bepotastine, but not hydroxyzine, may have already reached its maximum plasma concentration (C_max_) by the time of initial IRR onset.

A previous retrospective study suggested that bepotastine may suppress the incidence of IRRs occurring after 2 h of rituximab infusion more effectively compared with diphenhydramine (first-generation H1RA) [10]. Type I hypersensitivity, one of the mechanisms responsible for IRRs induced by rituximab, exhibits a bimodal response: an immediate response within 60 min following antigen exposure, and a late response that typically develops after 4–6 h, which primarily involves eosinophils [20, 21]. Although hydroxyzine antagonizes only the H1 receptor, bepotastine exhibits additional anti-allergic properties, including the inhibition of eosinophil infiltration, mast cell stabilization, and reduction of interleukin IL-5 production, which is responsible for recruiting eosinophils into the peripheral circulation [22]. This suggests that the efficacy of bepotastine at suppressing IRRs may be attributed, in part, to these additional anti-allergic properties. In the present study, we set the evaluation period for IRRs up to 4 h following the initiation of rituximab infusion, because our retrospective exploratory study indicated that the total rituximab infusion time was approximately 4 h. We enrolled both inpatients and outpatients, and considered a practical time frame for the evaluation. However, rituximab-associated IRRs occur more than 4 h after infusion initiation, including delayed reactions that may be clinically relevant [23]. Therefore, the present study’s restricted observation window may have underestimated the overall incidence of IRRs and the potential differences between the two antihistamines, particularly given the anti-allergic properties of bepotastine. In fact, several patients appeared to have IRRs (fever) after the observation period of 4 h, as recorded in their medical charts. Future studies should extend the observation period to 24 h or longer to more precisely assess the efficacy of the study drugs, particularly for delayed-phase IRRs. To maintain blinding in our study, the drugs were encapsulated in an opaque gelatin capsule. Considering that the mean disintegration time for these capsules in the fed state is 12 ± 4 min [24], we considered that the study drug may exhibit a delayed time to reach T_max_ and time to initial IRR onset. In addition, our study protocol allowed a 30–60 min window between premedication and rituximab infusion initiation, in accordance with clinical practice. Given the T_max_ values of two study agents, the 30-min variance may have influenced plasma concentrations at the time of infusion and the incidence or timing of IRRs. Future confirmatory studies may require stricter infusion timing protocols to evaluate the effect more precisely.

The degree of drowsiness caused by H1RA tended to be lower in the bepotastine group compared with that in the hydroxyzine group. This is consistent with a previous study in which the first-generation H1RA showed potent central nervous system effects [9]. In the present study, we set the evaluation reporting time for drowsiness at 90 min based on the average T_max_ of hydroxyzine and bepotastine; however, it is important to consider that drowsiness resulting from H1RA is influenced not only by T_max_ and T_1/2_, but also by their interaction with H1 receptors in the central nervous system [25, 26]. Therefore, to adequately assess drowsiness, additional observation points at a later time following rituximab infusion may be required.

Regarding safety, neither group experienced any severe adverse events or significant complications attributed to H1RA. Moreover, the median of the total infusion time in the bepotastine group was significantly shorter compared with that in the hydroxyzine group. However, this finding should be interpreted cautiously, as infusion duration was an exploratory endpoint and the study was not statistically powered to detect differences in this outcome. IRRs cause treatment interruptions and delays, and the administration of rescue medications is often required. This causes additional burdens to the patients and healthcare providers [27]. Bepotastine may benefit both patients and healthcare providers; thus, bepotastine may be the preferred premedication for IRRs with more efficacy and less drowsiness. However, because standard rescue care for IRRs was implemented according to the institution-specific protocol, differences in standard care may have influenced the total infusion duration of rituximab.

Several studies have examined the risk factors for the development of IRRs. Soluble IL-2 receptor levels, hemoglobin levels, bone marrow infiltration, and lactate dehydrogenase levels are considered risk factors [28–30]. In the present study, randomization was stratified by bone marrow infiltration. In this exploratory subgroup analysis, the total incidence of ≥ grade 2 IRRs in the bepotastine group tended to be lower compared with that in the hydroxyzine group among patients without bone marrow infiltration. In contrast, the total incidence of ≥ grade 2 IRRs was similar between the two groups in patients with bone marrow infiltration. These observational exploratory subgroup analyses should be investigated in larger, adequately powered studies.

The present study had several limitations. First, the small sample size precluded a definitive conclusion on the benefit of bepotastine; therefore, CIs were wide for the estimate of differences, indicating that the target estimation accuracy was not achieved in 40 cases. The wide CIs indicate substantial uncertainty in the estimated treatment effect, reflecting the limited precision associated with the small sample size. Therefore, these findings should be interpreted cautiously and regarded as hypothesis-generating. Adequately powered phase III trials will be necessary to confirm the observed numerical differences. Second, the incidence of late response IRRs after 4 h, following the initiation of rituximab infusion, was not formally evaluated. For a more precise assessment of the efficacy of the study drugs, extending the observation period to approximately 24 h or more may be required. Third, many second-generation H1RAs, other than bepotastine, are available in the marketplace. Considering that the IRR-suppressive effect of H1RAs may be associated with the relationship between T_max_ and timing of cytokine release, H1RAs with a shorter T_max_, such as olopatadine (1.0 h) [31] and levocetirizine (1.0 h) [32], may provide superior prophylactic efficacy compared with bepotastine (1.2 h). In addition, bilastine, desloratadine, and fexofenadine have lower central nervous system effects among the second-generation H1RAs [33–35]. Therefore, whether bepotastine is the most suitable rituximab premedication should be further evaluated.

## Conclusions

Bepotastine did not show significant superiority over hydroxyzine in preventing rituximab-induced IRRs due to the small sample size. Nevertheless, the exploratory study provides insight for further confirmatory studies.

## Abbreviations

CIs: Confidence intervals
CTCAE: National Cancer Institute Common Terminology Criteria for Adverse Events
FAS: Full analysis set
H1RA: Histamine H1 receptor antagonist
IL: Interleukin
IRRs: Infusion-related reactions
NHL: Non-Hodgkin lymphoma
PPS: Per-protocol set
R-CHOP: The regimen involving rituximab, cyclophosphamide, doxorubicin, vincristine, and prednisone
SAS: Safety analysis set
T_max_: Time to reach maximum plasma concentration
TNF: Tumor necrosis factor
T_1/2_: Half-life
VAS: Visual analog scale
